# Replication of Rift Valley Fever Virus in Amphibian and Reptile-Derived Cell Lines

**DOI:** 10.3390/pathogens10060681

**Published:** 2021-05-31

**Authors:** Melanie Rissmann, Matthias Lenk, Franziska Stoek, Claudia A. Szentiks, Martin Eiden, Martin H. Groschup

**Affiliations:** 1 Institute of Novel and Emerging Infectious Diseases, Friedrich-Loeffler-Institut, Insel Riems, 17493 Greifswald, Germany; melanie.rissmann@fli.de (M.R.); franziska.stoek@fli.de (F.S.); martin.eiden@fli.de (M.E.); 2 Department of Experimental Animal Facilities and Biorisk Management, Friedrich-Loeffler-Institut, Insel Riems, 17493 Greifswald, Germany; matthias.lenk@fli.de; 3 Department of Wildlife Diseases, Leibniz Institute for Zoo and Wildlife Research, 10315 Berlin, Germany; szentiks@izw-berlin.de

**Keywords:** Rift Valley fever phlebovirus, amphibians, reptiles, viral growth kinetics, primary cells

## Abstract

Rift Valley fever phlebovirus (RVFV) is a zoonotic arthropod-borne virus, which has led to devastating epidemics in African countries and on the Arabian Peninsula. Results of in-vivo, in-vitro and field studies suggested that amphibians and reptiles may play a role as reservoir hosts of RVFV, promoting its maintenance during inter-epidemic periods. To elucidate this hypothesis, we examined two newly established reptile-derived cell lines (Egyptian cobra and Chinese pond turtle) and five previously generated reptile- and amphibian-derived cell lines for their replicative capacity for three low- and high-pathogenic RVFV strains. At different time points after infection, viral loads (TCID_50_), genome loads and the presence of intracellular viral antigen (immunofluorescence) were assessed. Additionally, the influence of temperatures on the replication was examined. Except for one cell line (read-eared slider), all seven cell lines were infected by all three RVFV strains. Two different terrapin-derived cell lines (Common box turtle, Chinese pond turtle) were highly susceptible. A temperature-dependent replication of RVFV was detected for both amphibian and reptile cells. In conclusion, the results of this study indicate the general permissiveness of amphibian and reptile cell lines to RVFV and propose a potential involvement of terrapins in the virus ecology.

## 1. Introduction

Rift Valley fever phlebovirus (RVFV) is an arthropod-borne zoonotic virus of the family *Phenuiviridae*, genus Phlebovirus. The disease Rift Valley fever (RVF) has a considerable impact on livestock and human health and is endemic throughout extensive parts of Africa and on the Arabian Peninsula. The tripartite ambisense genome of the negative stranded RNA virus encodes the RNA-dependent RNA polymerase (L-segment), the two surface proteins Gn and Gc, as well as the nonstructural protein NSm (M-segment), the nucleoprotein NP and the nonstructural protein NSs (S-segment) [1].

Clinical manifestations of RVF in livestock are largely age- and species-dependent. Although only mild symptoms are found in adult ruminants and camels, so-called “abortion storms” can result in newborn fatalities of up to 100% [2]. Humans are primarily infected via contact to viremic animals and rarely by mosquito bites. Most human infections proceed with mild, flu-like symptoms. However, severe manifestations with meningoencephalitis, retinitis or hemorrhagic fever syndromes are observed in 1–2% of cases [3,4].

As an arthropod-borne disease, the emergence of RVF is dependent on the abundance of competent vectors. RVFV was found in more than 30 mosquito species of six genera, whereas *Aedes* spp. were found to be primary vectors for transmission and spread of the virus [5]. Their capability of transovarial transmission of RVFV defines them as a substantial variable of viral maintenance [6]. However, the additional existence of yet-unknown amplification hosts that may favor maintenance of RVFV during endemic cycles needs to be considered and has been repeatedly discussed [7,8,9,10,11,12]. Although numerous studies have been performed to define the reservoir host of RVFV, data are not conclusive for definite answers [12].

Various indications are found in literature that virus amplification occurs in amphibians and reptiles. Evidence of replication of diverse arboviruses has been repeatedly obtained in different reptiles and amphibians [13,14,15,16,17,18]. Although these species were assumed to be non-susceptible to RVFV in simple challenge studies in 1931 [19], field investigations in Kenya and Uganda found amphibians and reptiles to be a frequent source of blood meal for RVFV-competent mosquitoes [20,21]. An extensive screening of the in vitro susceptibility of various cell lines to RVFV already demonstrated that cells of the African clawed frog (*Xenopus laevis*) promote viral replication [22]. Moreover, a limited replication of RVFV has been found in agamas after experimental infection quite recently [23].

To gain a comprehensive insight into the general susceptibility, replication capacity and characteristics of RVFV infection of amphibians and reptiles in vitro, seven different cell lines of poikilothermic species origin were infected with three different low- and high-pathogenic RVFV strains. The viral growth kinetics were investigated by virus titration, genome quantification and immunofluorescence. Since temperature-dependent replication of viruses in reptiles has been repeatedly detected in vivo [24,25], this study also evaluated this potential influence specifically for RVFV.

## 2. Results

### 2.1. Generation of Primary Cell Lines

Primary cell lines of kidneys of an Egyptian cobra (*Naja haje*; MaKo) and a Chinese pond turtle (*Mauremys reevesii*; CDSK) were generated. CDSK was an outgrowth of explants, MaKo arose from trypsinized tissue. Growing cells displayed a homogenous spindle-shaped morphology. The species of origin for both cell lines were confirmed by their cytochrome B sequence with BLAST.

### 2.2. Virus Quantification

**Figure 1:**
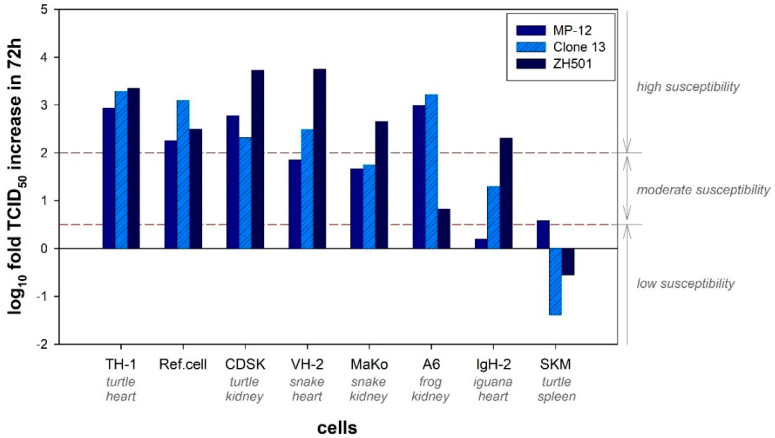
Increase in TCID_50_ within 72 h after RVFV infection. The increase in TCID_50_ from 0 hpi to 72 hpi (TCID_50_ 72 hpi–TCID_50_ 0 hpi) is depicted for every cell line and every virus strain. The average of three replicates is presented. Ref.cell: Reference cell lines (Vero76 for MP-12; BHK-21 Clone 13; VeroE6 for ZH501). Low susceptibility was defined as a log_10_ fold TCID_50_ increase in 72 h up to 0.5; moderate susceptibility up to 2.0 and high susceptibility from 2.0.

A varying amplification level was detected for the three virus strains in the seven different cell lines. MP-12 and Clone 13 were most efficiently amplified in TH-1 cells (Common box turtle), whereas the ZH501 strain showed strongest replication in CDSK cells (Chinese pond turtle) (Figure 1). The lowest susceptibility for MP-12 was seen in IgH-2 cells (Green iguana) and for Clone 13 and ZH501 in SKM-R cells (Red-eared slider). Although all three virus strains were amplified in the amphibian and reptilian cells in a comparable degree, ZH501 displayed the highest amplification activity (average of log_10_ fold increase from 0 h post infection (hpi) to 72 hpi: 3.25 log_10_ TCID_50_/mL), compared to Clone 13 (2.83 log_10_ TCID_50_/mL) and MP-12 (2.5 log_10_ TCID_50_/mL).

Except for SKM-R cells, all other cell lines were susceptible to all three strains of RVFV. A strong replication activity of all three strains was observed in TH-1 cells, while other cells best promoted ZH501 virus growth (CDSK, VH-2 (Russel’s viper)). The second highest amplification was observed in CDSK cells, although, compared to the other utilized cells, it promoted viral growth of Clone 13 the least. Albeit the replication of MP-12 was only moderate, VH-2 cells were also highly susceptible for RVFV infection. The three cell lines MaKo (Egyptian cobra), A6 (African clawed frog) and IgH-2 were moderately susceptible to RVFV, with low replication of ZH501 strain in A6 cells and MP-12 strain in IgH-2 cells. SKM-R cells were resistant to RVFV infections with Clone 13 and ZH501 and moderately susceptible to the MP-12 strain.

**Figure 2:**
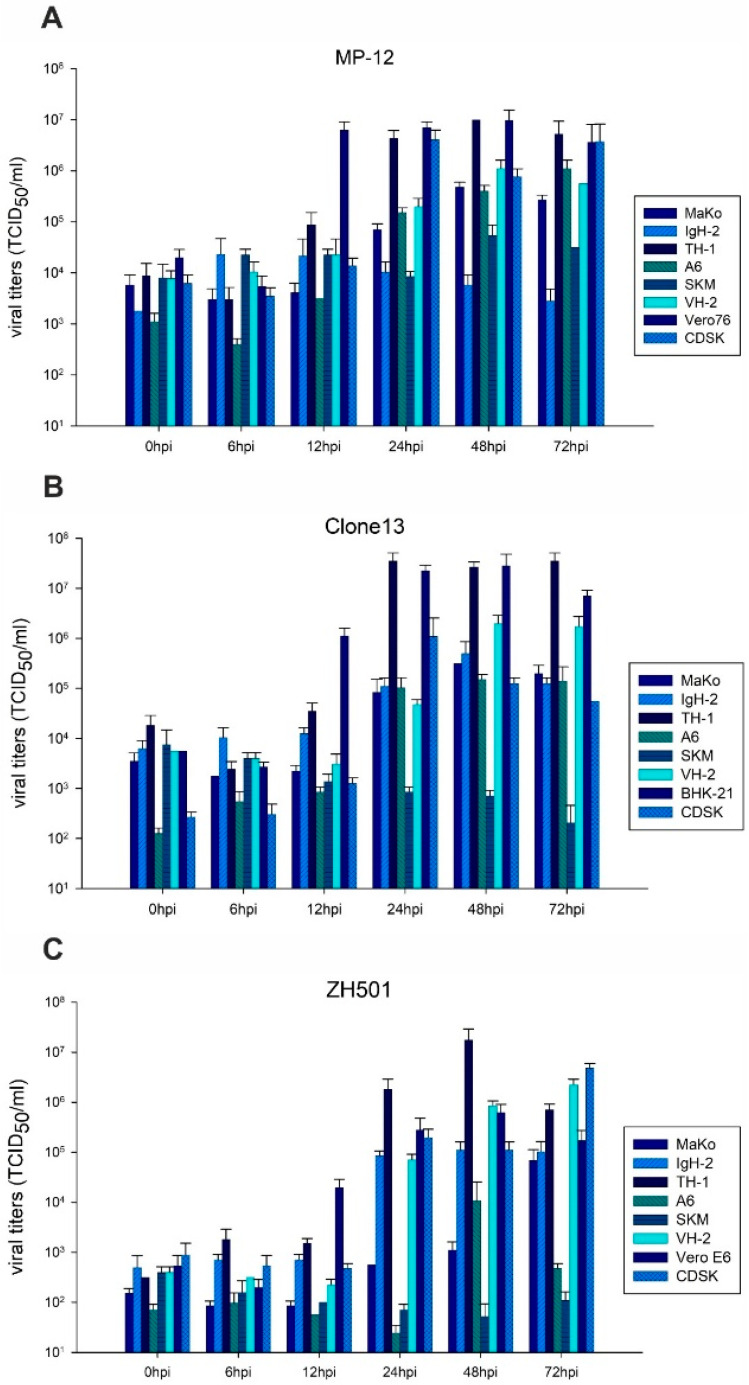
Viral replication. Viral replication of RVFV MP-12 (**A**), Clone 13 (**B**) and ZH501 (**C**) on reptile- and amphibian derived cell lines and corresponding reference cell line is presented. The average of three replicates per time point per cell line is depicted. Error bars indicate the standard deviation (SD).

An infection with the RVFV MP-12 strain resulted in virus infectivity peaks after 24 hpi (TH-1, CDSK) to 48 hpi (MaKo, A6, SKM-R, VH-2) (Figure 2A). The first increases in viral titers were observed at 12 hpi in highly susceptible cells (TH-1). Maximal titers for each cell line were ranging between 10^4.36^ and 10^7^ TCID_50_/mL. For the cell line IgH-2, no significant increase in viral replication was detected. The highest amplification was observed in TH-1 cells (10^7^ TCID_50_/mL at 48 hpi), with viral loads that were comparable to those of the reference cell line Vero76 (10^6.9^ TCID_50_/mL at 48 hpi). The two cell lines that were most susceptible to RVFV MP-12 infection (TH-1 and CDSK) also showed the earliest virus amplifications.

Peaks of Clone 13 replication were observed from 24 hpi (IgH-2, CDSK, MaKo, TH-1, A6) to 48 hpi (VH-2) (Figure 2B). Like for MP-12 infection, the earliest rise of viral titers was detected in TH-1 cells at 12 hpi. The maximal titers of Clone 13 were ranging between 10^5.1^ and 10^7.55^ TCID_50_/mL in the different cells. SKM-R was resistant to infection with Clone 13 and, compared to gained values at 0 hpi, even decreasing titers were detected. Although VH-2 cells showed a delayed onset of enhanced replication of Clone 13, the second highest viral loads were produced in these cells. The fastest and also most efficient replication of Clone 13 was detected in TH-1 cells (10^7.55^ TCID_50_/mL at 72 hpi), which was slightly higher than in the reference cell line BHK-21 (10^7.45^ TCID_50_/mL at 48 hpi).

The infection with the ZH501 strain led to viral peaks at 24 hpi (IgH-2), 48 hpi (TH-1, A6, VH-2) and 72 hpi (MaKo, CDSK) (Figure 2C). Compared to the two aforementioned attenuated strains, the increase in viral titers was delayed, i.e., detected about 24 h later in all ZH501-infected cells. Maximal titers for each cell line were ranging between 10^4.02^ and 10^7.24^ TCID_50_/mL. SKM-R cells were resistant to RVFV ZH501, as already observed for Clone 13. For both the cells A6 and TH-1, a drop of viral titers was observed after 72 hpi. However, the highest titers were again produced by TH-1 cells (10^7.24^ TCID_50_/mL at 48 hpi), which were significantly higher than those in the reference cell line Vero E6 (10^5.8^ TCID_50_/mL at 48 hpi). Comparably high titers that did exceed those of the reference cells (10^5.24^ TCID_50_/mL) were also produced after 72 hpi in CDSK (10^6.68^ TCID_50_/mL) and VH-2 cells (10^6.35^ TCID_50_/mL).

**Figure 3:**
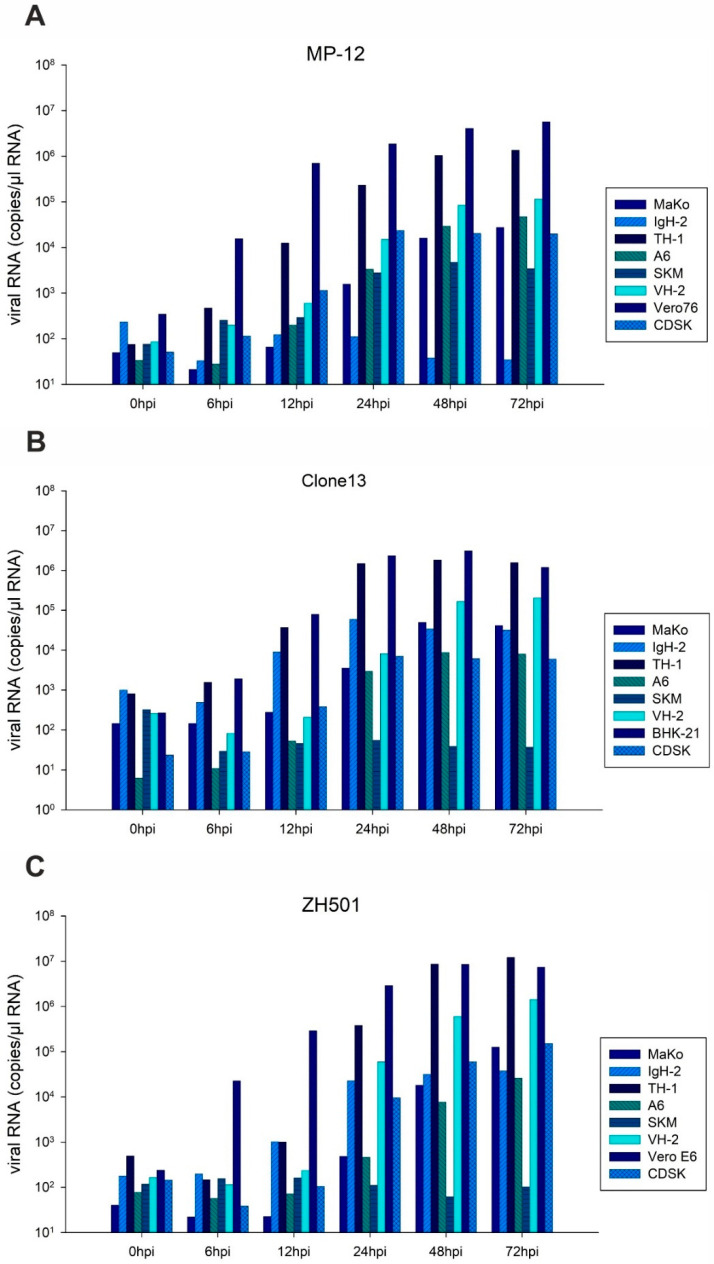
Genomic replication. Genomic replication of RVFV MP-12 (**A**), Clone 13 (**B**) and ZH501 (**C**) on reptile- and amphibian derived cell lines and corresponding reference cell line is presented. A pool of three replicates of cell culture supernatant was generated for RNA extraction for each time point and each cell line. Number of copies/µL RNA are depicted.

Viral RNA loads corresponded to the infectivity data (Figure 3). However, the increase in viral genomes was detected before viral loads increased. For MP-12 infection, differences between highly susceptible cells were not as evident as observed for the viral load in RT-qPCR. The reduced viral loads of ZH501 upon infection of TH-1 and A6 cells after 72 hpi was not observed for viral RNA. Comparing the three RVFV strains, the highest genome copy numbers were found for strain ZH501 (sum of genome amplification in all seven cell lines within 72 h (Sum_genome_): 10^7.33^ genome copies/µL RNA), compared to MP-12 (Sum_genome_: 10^6.86^ genome copies/µL RNA) and Clone13 (Sum_genome_: 10^6.49^ genome copies/µL RNA).

Although the cytopathogenic effect (cpe) generally correlated with viral titers, both parameters were not necessarily congruent for all cell lines (Supplementary Table S1). No cpe was observed in MP-12-infected SKM-R and CDSK cells, although CDSK cells did amplify virus to a high extent. A significant cpe was detected in all other utilized cells infected with MP-12 at 72 hpi. Although the cell line IgH-2 was only moderately susceptible, a cpe developed after 72 hpi. Generally, cell morphologies changed 24 h after viral titers increased. The infection with Clone 13 only caused cpe in TH-1 and in BHK-21 cells. With the exception of A6 and SKM-R, all ZH501-infected cell lines showed a cpe, starting already after 48 hpi. For VH-2, morphological differences were just detected 72 hpi.

Indirect immunofluorescence results generally mirrored those of viral replication (Table 1), with the exception that virus antigen production (nucleoprotein) preceded virus amplification in the cells. Highly permissive cell lines, such as TH-1, displayed pronounced fluorescence signals. The typical appearance of NSs filaments in the nucleus was also observed in all reptile cell lines (Supplementary Figure S1). Interestingly, no NSs could be detected in frog-derived A6 cells, independently of the used virus strain.

### 2.3. Validation of Temperature-Dependent Replication

**Figure 4:**
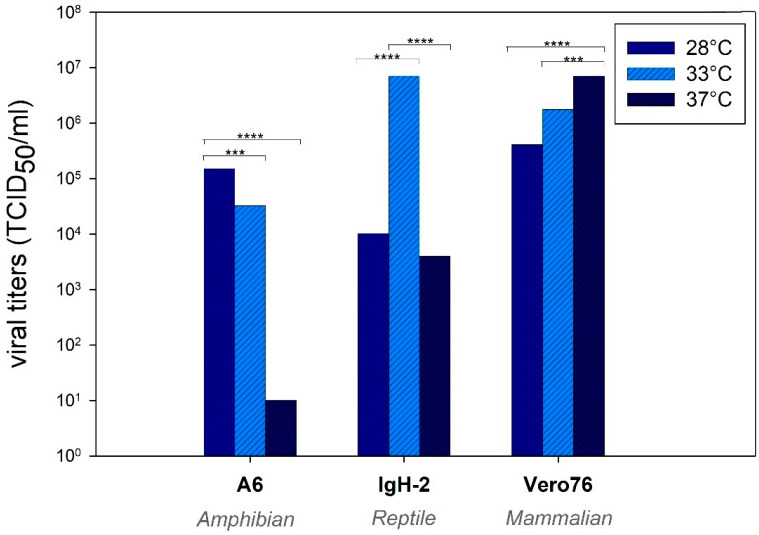
Temperature-dependent replication of RVFV MP-12 in mammalian, amphibian and reptile cells. The average of three replicates per time point per cell line is depicted. *** *p* < 0.001, **** *p* < 0.0001. One-way ANOVA; Bonferroni’s multiple comparison test.

The used cell lines showed a temperature-dependent growth (Supplementary Figure S2). MP-12 virus amplification was temperature-dependent in all three tested cell lines (Figure 4). Vero76 promoted the highest viral titers at 37 °C, and titers decreased almost linearly at lower temperatures. Interestingly, amphibian-derived A6 cells displayed the highest viral titers at 28 °C, while higher temperatures reduced the virus amplification. In contrast, reptile-derived IgH-2 cells allowed optimal virus growth at 33 °C.

## 3. Discussion

Identification of putative reservoir hosts would improve our understanding of the life cycle of RVFV, which is hardly understood. Definite reservoir hosts of RVFV have not been identified to date. Amphibians and reptiles are known to carry numerous potentially zoonotic arboviruses, such as members of the *Flaviviridae* and *Togaviridae* [13,14,15,16,17,18]. Their potential to act as reservoir hosts has been indicated through both field and in-vivo experiments, where infections were found to cause viral replication without clinical manifestations. Due to their enormous diversity and wide distribution in wetlands [13,27], they could also be ideal reservoir hosts for RVFV. Field investigations [20,21] already indicated a possible contribution of amphibians and reptiles to the maintenance of RVFV. In a previous study, our group demonstrated a limited susceptibility of agamas to RVFV in-vivo [23]. In this present study, we evaluated seven different amphibian- and reptile-derived cell lines in regards to their susceptibility to RVFV. Indeed, the results of such in-vitro infection experiments can only be transferred to the actual susceptibility of species and tissues to a limited extent and in-vivo infections with relevant animals must be carried out. However, with all due caution, in vitro infections can provide initial indications of species- and/or tissue-specific resistances or susceptibilities.

In order to assess the susceptibility of amphibians and reptiles, we analyzed five currently available cell lines for their capability to propagate RVFV. Moreover, we generated and tested two novel primary reptile-derived cell lines: Egyptian cobra (*Naja haje*, designated as MaKo) and Chinese pond turtle (*Mauremys reevesii*, designated as CDSK). These two new cell lines can also be valuable tools for the culture of reptile-associated viruses that might fail to grow in mammalian cell lines.

The results of our study demonstrate that amphibian- and reptile-derived cell lines strongly support efficient replication of three RVFV strains. Only the cell line SKM-R (red-eared slider) was not susceptible to the RVFV strains Clone 13 and ZH501 and showed only a very limited replication of MP-12 that might be explained by low-level amplification of stable remaining inoculum. All other cells amplified the RVFV strains and produced comparable, or in some cases even higher, virus loads than achieved in interferon-deficient reference cells (Vero76, Vero E6, BHK-21). Comparing all seven cell lines, the highest replication was detected in TH-1 (Common box turtle) and CDSK (Chinese pond turtle) cells. The VH-2 (Russel’s viper), A6 (African clawed frog), MaKo (Egyptian cobra) and IgH-2 (Green iguana) cells showed an intermediate replication.

Thus, cell lines derived from Chinese pond turtle and from common box turtle tissues (TH-1–heart; CDSK–kidney) were most susceptible. Both species belong to the family of terrapins and live in wetland habitats, therefore enabling a close interaction with mosquitoes. However, their distribution does not overlap with RVFV endemic areas. In contrast, the spleen cells of another terrapin (red-eared slider, SKM-R) were only mildly permissive to RVFV MP-12 and resistant to an infection with Clone 13 and ZH501. Unfortunately, no African terrapin cells are available for investigation to date, but a generation of such cells would be of great interest.

Previous studies demonstrated that, although having a 69% deletion of the NSs, Clone 13 is able to induce both a cpe and replication of virus in primary interferon-competent cells [28]. Clone 13 was used in this study to investigate whether the absence of NSs would significantly interfere with the replication of RVFV in reptile and amphibian cell lines. However, also in these cells, an efficient amplification of Clone 13 was observed, indicating that not solely the presence of NSs is the determinant for RVFV permissiveness in reptile- and amphibian-derived cells.

Utilized cells were derived from different tissues. No explicit tissue tropisms could be detected after infection with RVFV and both heart and kidney cells were highly permissive. However, since cells were additionally generated from different species, an in-depth comparison of tissue tropisms cannot be covered within this experimental setup.

A temperature-dependent replication was observed in three representative cell lines (Vero 76, A6 and IgH-2). Interestingly, the highest viral replication was found at the corresponding species-specific body temperature that is observed in nature and promotes best performance of the animals [29,30]. It can therefore be assumed that metabolism of each cell line is species-specific and an optimal temperature does also promote the highest virus replication. The temperature is additionally influencing the immune system of reptiles [24] and has a considerable impact on the severity of diseases and viral multiplication [25,31]. Moreover, it was demonstrated that the native cell growth was also temperature-dependent, being another possible explanation of enhanced viral replication at different temperatures. As already considered after in-vivo infection with RVFV [23], the ambient temperature of poikilothermic species seems to be of utmost importance for the course of RVFV infection and should be considered for future in-vivo and in-vitro experiments.

## 4. Materials and Methods

### 4.1. Cells and Viruses

**Figure 5:**
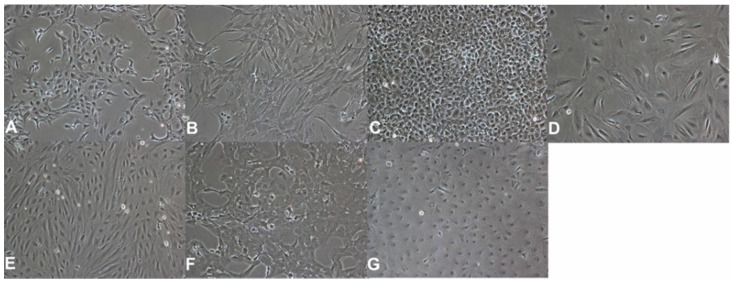
Morphology of used cell lines. Cells are depicted in a 100× magnitude. (**A**)—A6 cells (African clawed frog, *Xenopus laevis*). (**B**)—CDSK cells (Chinese pond turtle, *Mauremys reevesii*). (**C**)—IgH-2 cells (Green iguana, *Iguana iguana*). (**D**)—MaKo cells (Egyptian cobra, *Naja haje*). (**E**)—SKM-R cells (Red-eared slider, *Trachemys scripta elegans*). (**F**)—TH-1 cells (Common box turtle, *Terrapene carolina*). (**G**)—VH-2 cells (Russel´s viper, *Daboia russelii*).

Seven different primary cell lines from different tissues of amphibians and reptiles were utilized within this experimental setup (Figure 5, Table 2). Although liver cell lines would be of greatest interest according to the major tissue tropism of RVFV, their availability, especially for amphibians and reptiles, is limited. Since RVFV is furthermore known to have a pantropic tissue tropism in-vitro, all available cell lines independent of their tissue origin were used in this study. Cell lines originating from reptiles were acquired from the Collection of Cell Lines in Veterinary Medicine (CCLV, Friedrich-Loeffler-Institut, Germany), including TH-1 (Common box turtle; *Terrapene carolina*), IgH-2 (Green iguana; *Iguana iguana*), SKM-R (red-eared slider; *Trachemys scripta elegans*) and VH-2 (Russel’s viper; *Daboia russelii*). The amphibian cell line A6 (African clawed frog; *Xenopus laevis*) was received from the European Collection of Authenticated Cell Cultures (ECACC, Salisbury, United Kingdom). Kidneys of an Egyptian cobra (*Naja haje*) and a Chinese pond turtle (*Mauremys reevesii*) were kindly provided by the Leibniz Institute for Zoo and Wildlife Research (IZW, Department of Wildlife Diseases, Berlin, Germany) and primary cells were generated thereof. Briefly, fat and connective tissue were removed from the kidneys. Cleaned tissues were grossly chopped with scissors and scalpels and sterile phosphate-buffered saline (PBS) was added to centrifuge the crushed tissue (1000 rpm, 5 min, 20 °C). After discarding the supernatant, the process was repeated until the supernatant was clear. After washing, the issue pieces were further minced and washed as described before. The basic material was processed further by trypsination or was directly explanted into flasks (explant culture method). For the trypsination, a trypsin solution (Alsever’s Trypsin-Versen-solution; CCLV) was added to the tissue homogenate and the mixture was gently agitated with a magnetic spin for 45 min. After centrifugation (1000 rpm, 5 min, 20 °C), the cell pellet was dissolved in cell culture medium (Ham’s F-12 Nutrient Mixture and Iscove’s Modified Dulbecco’s Medium [1:1] with penicillin, streptomycin and amphotericin) and added to a ventilated T12.5 cell culture flask (BD Falcon, Erembodegem, Belgium) and incubated at 28 °C, 5% CO_2_. For the explant method, small pieces of minced and washed tissue were attached to the bottom of a ventilated T12.5 cell culture flask. After one hour, cell culture medium was carefully applied to the tissue. Cell morphology was closely monitored and after confluent growth cells were subcultivated and propagated for conservation in liquid nitrogen until further use.

To confirm the origin of applied cell lines on a species level, a cytochrome B gene analysis was performed. Initially RNA and DNA were extracted from cell pellets with the QIAamp Viral RNA Mini Kit (Qiagen, Hilden, Germany) according to the manufacturer’s instructions. Then, 2.5 μL of the resulting DNA was used as a template in a 22.5 μL amplification reaction. The PCR amplifications were conducted in a solution containing 5x GoTaq buffer (Promega, Madison, WI, USA), 25 mM MgCl2, 10 mM of dNTPs, 5 pmol of each primer and 1.25 units of Taq DNA polymerase (Promega). Primers used in the PCR are described by Kitano et al. [32]. PCR reactions were carried out as follows: 94 °C for 3 min, followed by 40 cycles consisting of 30 s at 94 °C, 30 s at 47 °C, and 60 s at 70 °C. The reaction was completed by incubation at 72 °C for 10 min. The PCR products were visualized by 1% agarose gel electrophoresis. PCR products were sent to Eurofins Genomics (Eurofins Genomics GmbH, Ebersberg, Germany) for DNA sequencing by the Sanger method using the amplification primers. Obtained sequences were analyzed with BLAST (NCBI) to identify the species.

The Rift Valley fever virus strains MP-12 (kindly provided by Richard Elliot, University of Glasgow, Centre for Virus Research, UK), Clone 13 (kindly provided by Friedemann Weber, Justus-Liebig University Gießen, Gießen, Germany) and ZH501 (kindly provided by Jeroen Kortekaas, University of Wageningen, Wageningen Bioveterinary Research, Lelystad, the Netherlands) were used for the infection of cells. The MP-12 was propagated on Vero76 cells (CCLV) and the Clone-13 on BHK-21 [C-13] cells (CCLV) under Biosafety level (BSL) 2 conditions. The ZH501 was propagated on VeroE6 cells (CCLV) under BSL-3 conditions. All virus strains were quantified with a 50% Tissue Culture Infective Dose (TCID_50_) assay on their corresponding reference cell line. Briefly, serial diluted RVFV strains were added to 90% confluent monolayers of cells. After incubation at 37 °C, 5% CO_2_ for six days, cells were fixed with neutral buffered formalin, stained with 1% crystal violet and the TCID_50_ was calculated as described by Spearman and Kaerber [33].

### 4.2. Experimental Setup

All cells were grown to about 90% confluence in Ø 35 mm dishes and were infected with a MOI of 0.1 of each virus strain, respectively. After virus adsorption for one hour at 28 °C and 5% CO_2_ (37 °C, 5% CO_2_ for reference cell lines), the infectious supernatant was removed and dishes were washed with sterile PBS. Then, 1.5 mL of corresponding cell culture medium with 2% fetal calve serum, penicillin and streptomycin were added to the dishes. Cells were incubated at 28 °C and 5% CO_2_, and three infected cell culture dishes of each cell line and each virus strain were frozen at 6, 12, 24, 48 and 72 h post infection (hpi). To determine a baseline of virus quantity, cells were additionally frozen immediately after infection (0 hpi). At every time point, cells were assessed for a cytopathogenic effect (cpe) and by immunofluorescence assay. After cell culture supernatants were harvested via freeze–thaw cycles, virus and genome loads were determined, utilizing a titration assay and quantitative real-time RT-PCR, respectively.

### 4.3. Virus Quantification

The TCID_50_ was assessed for triplicates of cell culture supernatants of every time point after infection. Briefly, serial diluted supernatants were added on 90% confluent monolayers of corresponding reference cells (Vero76 for MP-12; BHK-21 for Clone 13; VeroE6 for ZH501). After incubation at 37 °C, 5% CO_2_ for six days, cells were fixed with neutral buffered formalin, stained with crystal violet and the TCID_50_ was calculated.

RNA of cell culture supernatants was extracted using the QIAamp^®^ Viral RNA Mini Kit (Qiagen, Hilden, Germany) according to the manufacturer’s recommendations. The presence of RVFV-derived RNA was verified using a quantitative real-time RT-PCR (RT-qPCR) [34]. A synthetic RNA was utilized for quantification as described before [35].

### 4.4. Immunofluorescence Assay (IFA)

For IFA, cells were fixed with 4% paraformaldehyde (PFA). After washing with PBS, cells were permeabilized with 0.1% Triton X (Sigma-Aldrich, St. Louis, MI, USA) and 0.1 M Glycine (Carl Roth, Karlsruhe, Germany). Afterwards, cells were blocked (PBS containing 20% bovine serum albumin (Merck Millipore, Burlington, MA, USA), 0.2% Tween-20 (Sigma-Aldrich, St. Louis, MI, USA), 30% Glycerin (Carl Roth) and monoclonal antibodies against the glycoprotein N (encoded Gn3), the nucleoprotein (encoded NP3H3) and the nonstructural protein NSs (encoded NSs 5B7) [36,37] were added to the cells. After washing, a Cy3 (indocarbocyanin)-labeled goat anti-mouse IgG antibody (1:400; Jackson Immunoresearch, West Grove, PA, USA) and DAPI (1:20,000; Carl Roth) were added to the cells. After final washing, cells were evaluated by fluorescence microscopy.

### 4.5. Validation of Temperature-Dependent Replication

To examine the potential of divergent replication of RVFV in reptilian and amphibian cells depending on incubation temperature, the cells IgH-2 (Green iguana, representing cells of reptile origin and being lowly susceptible), A6 (African clawed frog, representing cells of amphibian origin) and Vero76 (Reference cells for MP-12 infections, mammalian origin) were infected with 0.1 MOI of RVFV MP-12 and incubated at 28 °C, 33 °C and 37 °C. The TCID_50_ was assessed 72 hpi. In this case, the potential of temperature-dependent replication, independent of the variable of used RVFV strain, was investigated. Therefore, MP-12 was chosen as representative of the three RVFV strains. Additionally, all three cell lines were grown and incubated at the indicated temperatures for 24, 48 and 72 h. The number of cells was counted at each time point to evaluate potential temperature-dependent differences in cell growth.

## Figures and Tables

**Table 1:** Presence of viral antigen (indirect immunofluorescence).

MP-12															
	6 hpi			12 hpi			24 hpi			48 hpi			72 hpi		
	Gn	NP	NSs	Gn	NP	NSs	Gn	NP	NSs	Gn	NP	NSs	Gn	NP	NSs
MaKo	-	-	-	-	-	-	-	+	-	+	+	-	+	+	+
IgH-2	-	-	-	-	-	-	+	+	+	+	+	+	+ *	+ *	+ *
TH-1	-	-	-	-	-	-	+	+	+	+	+	+	+	+	+
A6	-	-	-	-	+	-	-	+	-	-	+	-	-	+	-
SKM-R	-	-	-	-	+	-	-	+	+	+	+	-	+	+	-
VH-2	-	-	-	-	+	+	-	+	+	+	+	+	+	+	+
Vero 76	-	+	+	-	+	+	+	+	+	+	+	+	+	+	+
CDSK	-	-	-	-	+	-	+	+	+	+	+	+	+	+	+
Clone 13 ^1^															
	6 hpi		12 hpi		24 hpi		48 hpi		72 hpi						
	Gn	NP	Gn	NP	Gn	NP	Gn	NP	Gn	NP					
MaKo	-	-	-	+	-	+	+	+	+	+					
IgH-2	-	-	-	+	-	+	+	+	-	+					
TH-1	-	-	-	+	+	+	+	+	+	+					
A6	-	-	-	-	-	+	-	+	-	+					
SKM-R	-	-	-	-	-	-	-	-	-	-					
VH-2	-	-	-	-	-	+	-	+	-	+					
BHK-21	-	+	-	+	+	+	+	+	+	+					
CDSK	-	-	-	+	-	+	-	+	+	+					
ZH501															
	6 hpi			12 hpi			24 hpi			48 hpi			72 hpi		
	Gn	NP	NSs	Gn	NP	NSs	Gn	NP	NSs	Gn	NP	NSs	Gn	NP	NSs
MaKo	-	-	-	-	-	-	-	+	+	+	+	+	+	+	+
IgH-2	-	-	-	-	-	-	-	+	+	+	+	+	+	+	+
TH-1	-	-	-	-	+	-	+	+	+	+	+	+	+	+	+
A6	-	-	-	-	-	-	-	+	-	-	+	-	-	+	-
SKM-R	-	-	-	-	-	-	-	-	-	-	-	-	-	-	-
VH-2	-	-	-	-	+	-	+	+	+	+	+	+	+	+	+
Vero E6	-	+	+	+	+	+	+	+	+	+	+	+	+	+	+
CDSK	-	-	-	-	-	-	+	+	+	+	+	+	+	+	+

* Intensity of fluorescence was decreasing, compared to 48 hpi. ^1^ Clone 13 has a 69% deletion of NSs [26]. No NSs was detected.

**Table 2:** Origin and Characterization of used cell lines.

Cell Line	Species	Tissue	Origin	Medium
A6	African clawed frog (*Xenopus laevis*)	Kidney	ECACC ^1^	NCTC 109 mod.
CDSK	Chinese pond turtle (*Mauremys reevesii*)	Kidney	this study	Ham’s F12/IMDM (1:1)
IgH-2	Green iguana (*Iguana iguana*)	Heart	CCLV ^2^	MEM (E), NEA, 25 mM HEPES
MaKo	Egyptian cobra (*Naja haje*)	Kidney	this study	Ham’s F12/IMDM (1:1)
SKM-R	Red-eared slider (*Trachemys scripta elegans*)	Spleen	CCLV ^2^	Ham’s F12/IMDM (1:1)
TH-1	Common box turtle (*Terrapene carolina*)	Heart	CCLV ^2^	MEM (H+E), NEA
VH-2	Russell’s viper (*Daboia russelii*)	Heart	CCLV ^2^	MEM (E), NEA, 25 mM HEPES

^1^ European Collection of Authenticated Cell Cultures, Salisbury, United Kingdom. ^2^ Collection of Cell Lines in Veterinary Medicine, Friedrich-Loeffler-Institut, Insel Riems, Germany. H: Hank´s salts. E: Earle´s salts. NEA: Non-essential amino acids. HEPES: 4-(2-hydroxyethyl)-1-piperazineethanesulfonic acid.

## Data Availability

The data presented in this study are available in Rissmann, M.; Lenk, M.; Stoek, F.; Szentiks, C.A.; Eiden, M.; Groschup, M.H. Replication of Rift Valley Fever Virus in Amphibian and Reptile Derived Cell Lines. Pathogens 2021 and corresponding supplementary materials.

## Supplementary Materials

The following are available online at https://www.mdpi.com/article/10.3390/pathogens10060681/s1, Figure S1: Immunofluorescence assay; Figure S2: Temperature-dependent growth of cell lines Vero76, IgH-2 and A6; Table S1: Cytopathogenic effects.
