# Supplementary material for: Replication of Rift Valley Fever Virus in Amphibian and Reptile-Derived Cell Lines
Source: Pathogens. 2021 May 31;10(6):681. doi: 10.3390/pathogens10060681 (PMC8228813; doi:10.3390/pathogens10060681)
Supplement: Supplementary file 1 [file pathogens-10-00681-s001.zip › Table S1.pdf]

Table S1. Cytopathogenic effects

MP-12

	MaKo	*TH-1*	*IgH-2*	*A6*	*SKM*	*VH-2*	*Vero76*	*CDSK*
6hpi	-	-	-	-	-	-	-	-
12hpi	-	-	-	-	-	-	-	-
24hpi	-	-	-	-	-	-	-	-
48hpi	-	-	+	+	-	-	+	-
72hpi	+	+	+	+	-	+	+	-

Clone 13

	MaKo	*TH-1*	*IgH-2*	*A6*	*SKM*	*VH-2*	*BHK-21*	*CDSK*
6hpi	-	-	-	-	-	-	-	-
12hpi	-	-	-	-	-	-	-	-
24hpi	-	-	-	-	-	-	-	-
48hpi	-	-	-	-	-	-	-	-
72hpi	-	+	-	-	-	-	+	-

ZH501

	MaKo	*TH-1*	*IgH-2*	*A6*	*SKM*	*VH-2*	*VeroE6*	*CDSK*
6hpi	-	-	-	-	-	-	-	-
12hpi	-	-	-	-	-	-	-	-
24hpi	-	-	-	-	-	-	-	-
48hpi	-	+	+	-	-	-	+	+
72hpi	-	+	+	-	-	+	+	+

Cytopathogenic effects were evaluated at the according time points after infection before petri dishes were frozen. Infected cells were compared to corresponding mock-infected cells, that were treated and kept in the same way like infected cells.
