# Supplementary figures and images for: Replication of Rift Valley Fever Virus in Amphibian and Reptile-Derived Cell Lines
Source: Pathogens. 2021 May 31;10(6):681. doi: 10.3390/pathogens10060681 (PMC8228813; doi:10.3390/pathogens10060681)

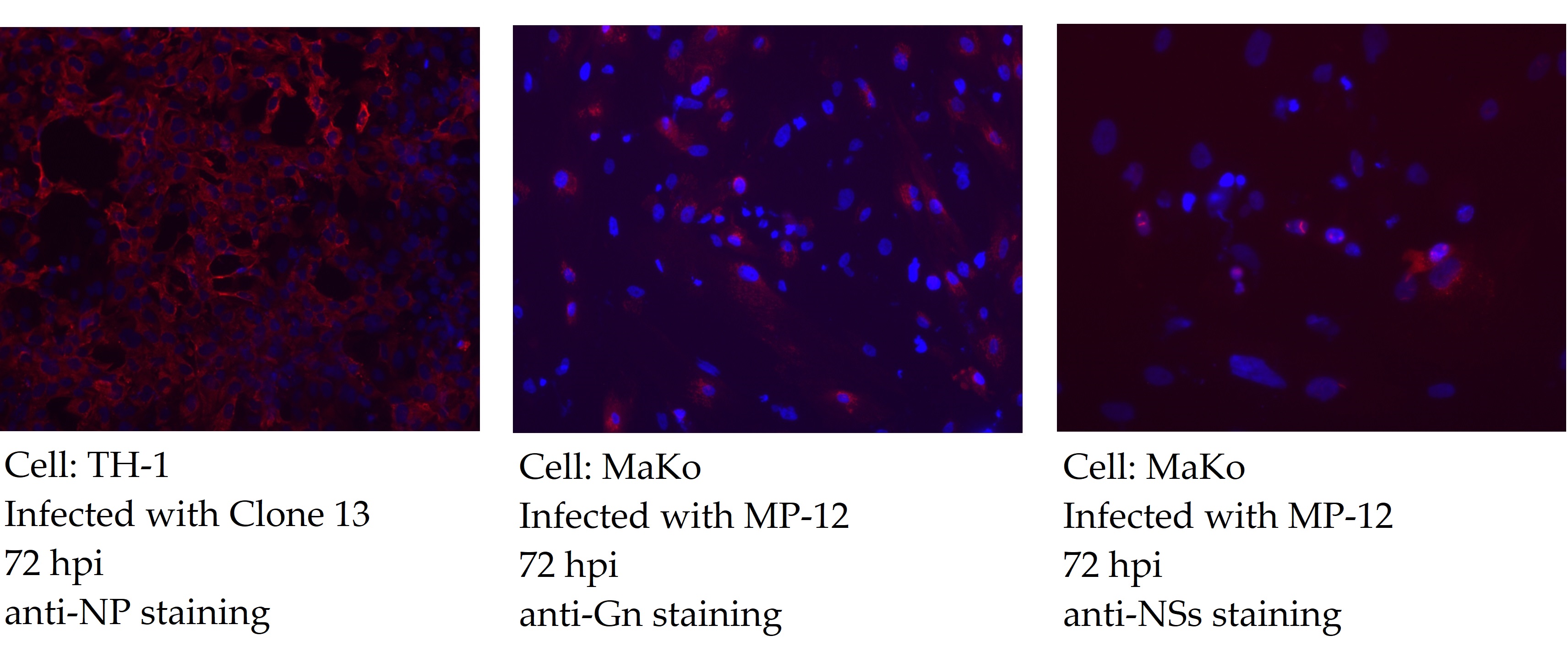

Supplement: Supplementary file 1 [file pathogens-10-00681-s001.zip › Figure S1.jpg]

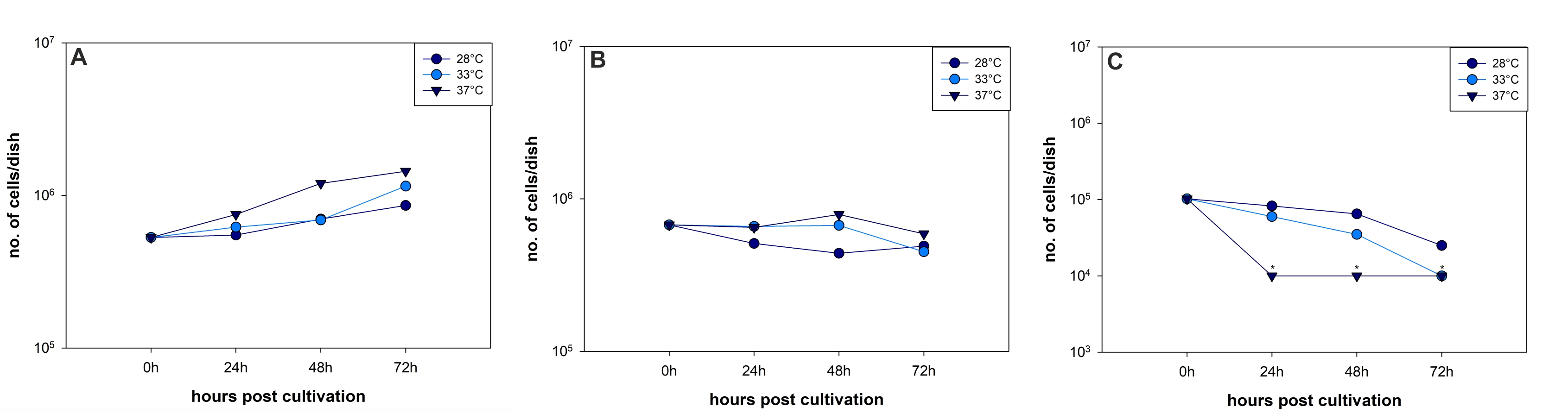

Supplement: Supplementary file 1 [file pathogens-10-00681-s001.zip › Figure S2.jpg]
